# Warm-season annual forages in forage-finishing beef systems: II. Animal performance and carcass characteristics

**DOI:** 10.1093/tas/txz181

**Published:** 2019-12-03

**Authors:** Deidre D Harmon, Dennis W Hancock, R Lawton Stewart, Jenna L Lacey, Robert W Mckee, John D Hale, Chevise L Thomas, Elyse Ford, Jacob R Segers, Chris D Teutsch, Alexander M Stelzleni

**Affiliations:** 1 Dept. of Crop and Soil Sciences, University of Georgia, Athens, GA; 2 Dept. of Animal and Dairy Science, University of Georgia, Athens, GA; 3 Dept. of Animal and Dairy Science, University of Georgia, Tifton, GA; 4 Dept. of Plant and Soil Sciences, University of Kentucky, Princeton, KY

**Keywords:** carcass, forage-finished beef, performance, summer annuals

## Abstract

More information on expected animal performance and carcass traits of forage-finished steers grazing warm-season annual forages is needed. To achieve this objective, a grazing trial was conducted in 2014, 2015, and 2016 (70, 63, and 56 d, respectively), with variation in length of grazing based on forage availability. Sixteen pastures (0.81 ha) were assigned to 1 of 4 forage treatments in a randomized complete block design. Forage treatments were brown midrib sorghum × sudangrass (BMR; Sorghum bicolor var. bicolor*bicolor var. sudanense), sorghum × sudangrass (SS), pearl millet [PM; *Pennisetum glaucum* (L.)R.Br.], or pearl millet planted with crabgrass [PMCG; *Digitaria sanguinalis* (L.) Scop.]. Each year, British-cross beef steers (*n* = 32; 3 y average: 429 ± 22 kg) were stratified by weight and randomly assigned to 1 of the 16 pastures for forage finishing. Each pasture was subdivided into two 0.405-ha paddocks for rotational stocking and a put-and-take stocking method was used to maintain a forage allowance of 116 kg forage dry matter/100 kg body weight (BW). Shrunk body weight and ultrasonically measured carcass composition were recorded at the initiation, middle, and end of each grazing season. Steers were harvested once forage availability became limited and chilled carcasses (24 h) were evaluated for yield grade and quality grade attributes. Statistical analysis was conducted using the GLIMMIX procedure in SAS 9.4 (Cary, NC) with main effects of treatment, year, and the interaction. Pasture and block were considered random effects while date was assessed as a main effect when applicable. Daily stocking densities were greater (*P* < 0.04) for SS than PMCG in the first 20 d of 2014 and 2015. Forage treatment did not affect (*P* > 0.17) total gain, total average daily gain, or body weight at any time point. Ultrasound composition traits of loin muscle area, 12th rib fat thickness, intramuscular fat, and rump fat were impacted (*P* < 0.01) by scanning date. No differences (*P* > 0.08) in forage treatments were observed for carcass characteristics associated with yield grade or quality grade. The findings suggest that forage-finished cattle during the summer months on BMR, SS, PM, and PMCG perform similarly, giving producers the option to use the most economical or practical forage type for their production system.

## INTRODUCTION

In the United States, the majority of beef cattle are finished in confined animal feeding operations on grain-based rations. These operations have been readily adopted because of their reduced production period (Hoveland and Anthony, 1977), decreased overall costs, increased efficiency (Mathews Jr. and Johnson, 2013), and ability to produce a uniform, high-quality beef product (Crouse et al., 1984; Garmyn et al., 2010). Although consumers have become accustom to the taste of grain-fed beef, there is a growing interest in forage-finished beef products. The interest in forage-finished beef products has primarily been stimulated by a general aversion to confined animal feeding systems, and reports that grass-fed beef is a healthier alternative and contains an altered fat content and fatty acid profile when compared to grain-fed beef (Duckett et al., 2009). As a result, it has been reported that consumers are willing to pay a premium for grass-finished beef products (Darby et al., 2006; Lacy et al., 2007).

In the southeastern United States, grass-finishing cattle during summer months can be difficult due to the combination of heat stress, unpredictable weather, and the characteristically high portion of indigestible fiber found in warm-season perennial forage species. Although forages such as bermudagrass (*Cynodon dactylon* L. Pers.) and bahiagrass (*Paspalum notatum* Flugge) produce high forage yields and are well suited for cow/calf operations, their nutritive value is typically not high enough to produce desirable rates of lean and adipose tissue growth needed for finishing cattle (Schmidt et al., 2013). Consequently, providing a year-round supply of grass-finished beef is challenged by an inability to finish cattle in the summer months.

In contrast, warm-season annual species such as sorghum × sudangrass hybrids [*Sorghum bicolor* (L.) × *S. Arundinaceum* (Desv.)], pearl millet (*Pennisetum glaucum* (L.)R.Br.), and crabgrass [*Digitaria sanguinalis* (L.) Scop.] are high in nutrient value and are high yielding, potentially enabling adequate animal performance in summer forage-finishing programs. Although an abundance of literature has been published comparing grain vs. forage-finished beef (Mandell et al., 1998; Leheska et al., 2008; Daley et al., 2010), there is little information regarding the effects of varying forage species on animal performance and carcass characteristics, especially studies comparing warm-season annual forage systems. In one of the few published studies, Schmidt et al. (2013) compared carcass characteristics of steers grazing 5 forage species, including both perennial and annual forages as well as a mix of cool- and warm-season grass and legume species. Although only 1 warm-season annual grass was included in this study, authors reported a forage species effect on both cattle performance and carcass characteristics, with pearl millet pastures producing average daily gains (ADG) over 0.5 kg/d and quality grades comparable to steers grazing alfalfa. In Canada, it was reported that sorghum × sudangrass could produce gains in steers of 0.97 to 1.18 kg/d. However, there has been no published research comparing sorghum × sudangrass and pearl millet forage systems in grass-finishing operations in the southeastern United States. Therefore, the objective of this study was to evaluate the animal performance, carcass characteristics, and beef quality of 4 warm-season annual forage systems for summer forage finishing of beef steers in the Southeast.

## MATERIALS AND METHODS

The experimental procedures were reviewed and approved by the University of Georgia Institutional Animal Care and Use Committee (IACUC approval number A2014 05-002).

### Forage Treatments

A 3-yr forage-finishing trial was conducted in the summers of 2014, 2015, and 2016 to determine the effects of forage treatment on animal performance and carcass merit. In a randomized complete block design with 4 replications, forage treatments of sorghum × sudangrass (SS), brown midrib sorghum × sudangrass (BMR), pearl millet (PM), and a mixture of pearl millet and crabgrass (PMCG; *D. sanguinalis*) were assessed. The trial consisted of sixteen 0.81-ha pastures located at the University of Georgia, Department of Animal and Dairy Science Eatonton Beef Research Unit in Eatonton, GA (33°24′N, 83°28′W; elevation 163 m). Pastures were blocked based on previous use, soil type, and topography, and forage treatments were randomly assigned within each block.

Forage treatments were planted into chemically burned (Helosate Plus; HELM Agro US, Inc., Tampa, FL) pastures each year on or about the 15 of May using a no-till drill (Haybuster 107; Haybuster, Jamestown, ND). Sorghum × sudangrass (cv. “Sugargrazer” in 2014 or cv. “AS5201” in 2015 and 2016; Alta Seeds, Irving, TX) and BMR (“Honey Graze” in 2014; Arrow Seed Co., Broken Bow, NE; or “AS6201” in 2015 and 2016; Alta Seeds, Irving, TX) were planted at 22.4 kg/ha and at a soil depth of 2.54 cm. The use of different SS and BMR varieties in 2014 was due to the lack of availability of the desired AS5201 and AS6201 varieties. In 2014, selected varieties were chosen as a result of their similar performance in the University of Georgia’s Statewide Variety Testing Program (Gassett et al., 2014) and is unlikely that changing varieties resulted in any confounding effects. Pearl millet (cv. “Tifleaf III”; Coffey Forage Seeds, Inc., Plainview, TX) was seeded at 16.8 kg/ha and at a soil depth of 1.27 cm, and the PM (cv. “Tifleaf III”) plus CG (cv. “Red River”; R.L. Dalrymple Farm, Thomas, OK) mixture was planted simultaneously at 11.2 kg/ha at 1.27 cm and 5.6 kg/ha at 0.64 cm, respectively. Crabgrass was planted in a 1:1 ratio with sand to reduce static cling and allow a consistent flow of crabgrass from the small seed box through the drop tubes.

During the spring of all trial years, soil core samples were taken from each pasture and analyzed for nutrient deficiencies. Based on soil core samples in 2014, it was recommended that 17-17-17 granular fertilizer be applied to all pastures at a rate of 448 kg/ha. Soil test from 2015 and 2016 did not indicate a need for phosphorus and potassium fertilizer and thus, it was not applied. Additionally, liquid nitrogen fertilizer (“19E”; R.W. Griffin, Attapulgus, GA; or 32% UAN) was applied at a rate of 45 kg/ha of N on day 30 and 34 in 2014 and 2015, respectively, and a reduced rate of 34 kg/ha of N was applied on day 37 in 2016 due to drought conditions and the concern of nitrate accumulation in drought-stressed plants. These amounts were applied to the one-half of the pasture that was resting, with the second half of each pasture receiving nitrogen fertilization approximately 7 to 14 d thereafter.

### Cattle Management

Each year, 32 angus-cross steers (3 y average: 429 ± 22 kg) from the Department of Animal and Dairy Science Eatonton Beef Research Unit were utilized. Steers engaged in this study were from the same herd and breeding season each year. Upon fall weaning at approximately 7 mo of age, steers were backgrounded on stockpiled, mixed-grass pastures that consisted primarily of bermudagrass (*Cynodon dacylon*) and tall fescue (*Festuca arundinacea* Schreb). In November of each year, yearling steers were sent to the University of Georgia’s, Georgia Mountain and Research Education Center, Blairsville. Here, they grazed stockpiled tall fescue and bermudagrass for 30 d, then fed a corn silage-based ration for approximately 100 d. In March of each year, steers were returned to the Eatonton Beef Research Unit where they grazed cool-season annual forages of ryegrass (*Lolium multiflorum* Lam) and crimson clover (*Trifolium incarnatum* L.), or tall fescue until the initiation of the summer annual grazing trial.

One week prior to the initiation of grazing, steers were weighed, stratified by BW, and randomly assigned to 1 of 4 forage treatments. Grazing was initiated on June 25 in 2014 and 2015, and June 29 in 2016. Upon initiation of the grazing trial, steers were fasted for 12 h, weighed, and ultrasound measurements were made for body composition by a trained ultrasound technician using an Aloka 500V with a 17-cm, 3.5-MHz transducer (Aloka Inc., Tokyo, Japan). Real-time ultrasound was used to estimate carcass measurements upon the initiation (day 0), middle (day 34), and on the last day (September 3, August 27, and August 31 of 2014, 2015, and 2016, respectively) of the grazing trial during each year. Ultrasound measurements of ribeye area (uLM) at the 12th and 13th rib juncture, 12th rib fat thickness (uFT), rump fat thickness (uRFT), and intramuscular fat percentage (uIMF) were collected from the right side of each steer and analyzed using Beef Image Analysis Feedlot software (Designer Genes Technologies Inc., Harrison, AR).

All steers were supplied with ad libitum access to shade, water, and mineral (McNess Bova Breeder 6; Furst McNess Co., Cordele, GA; Table 1) throughout the trial. Each 0.81-ha pasture was subdivided into two 0.405-ha pastures with temporary fencing and rotationally stocked. Rotational decisions were made based on forage availability (measured biweekly) and residual height of pastures adequate for optimal regrowth potential (Allen et al., 2011). Put-and-take stocking was also used to maintain forage DM availability of 116 kg forage DM/100 kg BW and steers that were added or removed were from the same contemporary group as the 32 test steers. All put-and-take steers were fasted for 12 h prior to being weighed and added to pastures.

**Table 1.** Composition of free-choice mineral^1^

Ingredient	Guaranteed analysis
Calcium, %	13.2
Phosphorus, %	6.1
NaCl, %	20.0
Magnesium, mg/kg	2.6
Zinc, mg/kg	9,000.0
Manganese, mg/kg	6,500.0
Copper, mg/kg	3,000.0
Iodine, mg/kg	184.5
Cobalt, mg/kg	45.0
Selenium, mg/kg	39.0
Vitamin A, IU/kg	661,387.0
Vitamin D-3, IU/kg	66,139.0
Vitamin E, IU/kg	1,322.0

^1^McNess Bova Breeder 6 (Furst McNess Co., Cordele, GA).

Once put-and-take steers were removed from the pastures, gains were determined by taking the average ADG for the tester steers in the respective pasture and multiplying that by the number of days a put-and-take steer spent grazing a specific pasture. The BW of the put-and-take steers, in combination with the test steer BW were both used in the calculation of stocking density. Additional information on animal grazing and forage management is available in Harmon et al. (2019).

### Carcass Data Collection

Once forage DM availability became limiting, steers were transported to the Department of Animal and Dairy Science Meat Science Technology Center in Athens, GA. End dates were September 3, August 27, and August 31 in 2014, 2015, and 2016, resulting in steers grazing for 70, 63, and 56 d, respectively. Steers were held in outside, covered pens and were given ad libitum access to fresh water for 12 h prior to being harvested under United States Department of Agriculture federal inspection. Steers were slaughtered in 2 separate but equal groups approximately 48 h apart in order to accommodate the facilities daily slaughter capacity. Immediately prior to slaughter, BW was collected on each animal. Following hide removal, carcasses were split, weighed (hot carcass weight), and washed with warm water followed by a 4.5% lactic acid wash before being chilled for 24 h at −2°C. Following the chilling period, the right side of each carcass was ribbed between the 12th and 13th rib junction and allowed to bloom for approximately 30 min before carcass yield and quality measurements were taken. Variables measured included 12th rib fat thickness, LM area, percent kidney, pelvic, and heart fat (KPH), marbling score, and skeletal, lean, and overall maturity. In addition, both objective and subjective lean and fat color measurements were taken. Objective measurements of lean were taken in a 50-mm-diameter area, in triplicate, on the exposed logissimus muscle with a Hunter-Lab Miniscan EZ (CR-310; Hunter Associates Laboratory, Inc.; Reston, VA) with illuminate A at a 10° viewing angle, 2.54 cm aperture. Prior to each use the colorimeter was calibrated to white, black, and saturated red tiles. Objective fat color measurements were taken near the posterior rib and on the same carcass side as for lean color. Subjective color of lean was measured on a scale of 1 through 8 with a 1 representing extremely dark red and an 8 representing light cherry red. Subjective color of fat was measured on a scale of 1 (white) through 5 (yellow). Yield grades were also calculated for each carcass using standard methods (USDA-AMS, 1997).

### Statistical Analysis

All statistical analyses were conducted using the GLIMMIX procedure in SAS 9.4 (SAS Inst. Inc., Cary, NC) to determine interaction and main effects of treatment and year. When applicable, day was used as a main effect and analyzed with interactions. Pasture and block were considered random effects and an alpha level of 0.05 was used to determine significance of main effects, with least squares means separated by pairwise comparisons using a *t*-test.

## RESULTS AND DISCUSSION

### Total Stocking Capacity

Stocking densities were affected (*P* < 0.01) by an interaction of year, treatment, and day. Thus, weekly stocking data were analyzed and presented by year and day (Table 2). Forage treatment affected (*P* < 0.01) stocking densities on day 20 and before in the 2014 grazing year. Upon initiation of the grazing trial in 2014, SS and BMR carried a greater (*P* < 0.01) stocking density than PM and PMCG pastures. On day 6 and 13 in 2014, SS carried more (*P* < 0.01) kg/ha of animal than BMR, and the BMR had a greater (*P* < 0.05) stocking density than PM or PMCG. In the first 2 wk of the trial, BMR pastures contained a greater (*P* < 0.01) stocking density than the pearl millet treatments, though this effect disappeared (*P* > 0.10) 20 d into the 2014 grazing trial. Ample soil moisture and a more rapid forage growth in the SS and BMR pastures in 2014 (Harmon et al., 2019) resulted in a greater need to put increased stocking pressure on those pastures. However, after the initial challenge of keeping up with the flush of early season forage productivity in the SS and BMR pastures, grazing pressure became similar across treatments. This did not occur in 2015 and the effect was generally muted in 2016 as a function of drier conditions and less rapid, early season forage growth, indicating that moisture availability may be a larger driver in the establishment and early growth of sorghum × sudangrass than pearl millet or crabgrass. Additional information on drought management, forage mass, and forage nutritive value is available in Harmon et al. (2019). In 2016, a treatment effect (*P* < 0.04) was found at day 0, 6, 13, and 20. Pearl millet and crabgrass mixed pastures contained the lowest stocking density at day 0 and 6 compared to the other forage treatments. On day 13 and 20, SS had a greater (*P* < 0.01) stocking density than PMCG, with both BMR and PM as intermediates. Results found in this study indicate that BMR and SS pastures can maintain higher levels of stocking densities early in a grazing program when conditions allow forage growth rates to reach their potential. However, the grazing management challenges associated with early, rapid forage growth of all treatments may be alleviated with multiple planting dates in an attempt to spread out the forage distribution and reduce fluctuations in stocking densities. In a 2-yr forage trial, Fontaneli et al. (2001) suggested that the production period of sorghum × sudangrass and pearl millet could be increased by planting on 2 seeding dates with 3 to 6 wk between plantings. Authors also reported that for each day planting was delayed after the initial planting date, total dry matter yield decreased at a rate of 25 to 30 kg/ha/d the first year and 23 to 36 kg/ha/d the second year. Similarly, Hancock and Durham (2010) found that pearl millet planted after late April declined in total DM yields of 41 to 88 kg/ha/d. Authors noted that the more rapid declines in their research compared to previous literature were the result of moisture deficiencies and inconsistencies in rainfall patterns compared to studies where irrigation was used.

**Table 2.** Daily stocking densities (kg of BW/ha) of sorghum × sudangrass (SS), brown midrib sorghum × sudangrass (BMR), pearl millet (PM), and pearl millet and crabgrass mixture (PMCG) pastures in 2014, 2015, and 2016 at the University of Georgia, Department of Animal and Dairy Science Eatonton Beef Research Unit in Eatonton, GA

Year/day	Forage treatment, kg of BW/ha				SEM	*P*-value
	SS	BMR	PM	PMCG		
2014						
0	3,998^a^	4,036^a^	2,158^b^	2,157^b^	38.9	<0.01
6	6,987^a^	4,086^b^	2,177^c^	2,187^c^	223.7	<0.01
13	9,719^a^	6,175^b^	4,099^c^	4,799^c^	311.8	<0.01
20	8,918^a^	6,265^b^	4,404^b^	5,626^b^	723.6	0.01
27	7,036	5,634	4,704	5,468	633.6	0.11
34	3,223	3,212	4,488	4,536	932.0	0.61
41	2,711	2,499	3,581	3,309	484.4	0.38
48	2,738	2,530	2,791	2,493	191.6	0.63
55	2,766	2,561	2,831	2,517	194.8	0.62
62	2,793	2,593	2,872	2,540	198.2	0.61
69	2,821	2,624	2,912	2,563	201.8	0.60
2015						
0	5,382	5,325	5,695	4,684	355.2	0.30
6	4,643^b^	5,396^ab^	5,760^a^	4,738^b^	391.8	0.05
13	3,626	3,606	3,526	3,267	402.0	0.79
20	3,951	4,187	3,842	3,566	310.2	0.20
27	3,730	3,667	3,610	3,612	354.4	0.99
34	2,325	2,348	2,315	2,306	14.9	0.28
41	2,332	2,369	2,322	2,322	13.4	0.10
48	2,340	2,390	2,328	2,337	15.0	0.06
55	2,347	2,411	2,335	2,353	18.8	0.07
62	2,354	2,432	2,341	2,368	23.9	0.10
2016						
0	3,048^a^	3,246^a^	2,744^a^	2,020^b^	268.1	0.01
6	3,331^a^	3,303^a^	2,790^a^	2,059^b^	226.3	<0.01
13	6,047^a^	4,473^ab^	4,504^ab^	2,680^b^	788.5	0.04
20	6,133^a^	4,367^ab^	4,363^ab^	2,735^b^	760.7	0.04
27	2,187	2,183	2,392	2,493	176.1	0.54
34	2,196	2,197	2,189	2,179	27.2	0.96
41	2,240	2,248	2,234	2,235	28.6	0.98
48	2,283	2,300	2,279	2,291	30.5	0.96
55	2,325	2,351	2,324	2,348	33.1	0.90

^a–c^Means within a row without a common superscript differ (*P* < 0.05)

### Animal Performance

Body weight and ADG differed by year (*P* < 0.01; Table 3), and this was expected given the drastic swing in moisture availability. However, there was no interaction of treatment by year (*P >* 0.15) or treatment (*P >* 0.16) on body weight or ADG. Steers were heavier (*P* < 0.01) at initiation of the grazing trial in 2014 and 2015 compared to 2016 (440, 437, and 411 kg, respectively), indicating steers in those years were more advanced in their growth and may have been on an increased nutritional plane prior to the start of the grazing trial. Consequently, steers in 2014 and 2015 were also heavier (*P* < 0.01) at the middle of the grazing trial when compared to steers in 2016 (466, 470, and 450 kg, respectively). However, this effect may also be attributed to the timely precipitation events that occurred after emergence and early in the grazing trial in those 2 yr but was not observed in 2016. Final BW was greater (*P* < 0.01) in 2014 than in 2015 and 2016 (496, 481, and 474 kg, respectively) and may be reflective of the mild climate in combination with the increased number of grazing days in 2014.

**Table 3.** Least squares means for growth performance of forage-finished steers pastured on sorghum × sudangrass (SS), brown midrib sorghum × sudangrass (BMR), pearl millet (PM), or a mixture of pearl millet and crabgrass (PMCG) during a forage-finishing trial conducted during the summers of 2014 to 2016 at the UGA Animal and Dairy Science Department’s Beef Research Unit near Eatonton, GA

Item	Forage treatment				SEM	Effect		
	SS	BMR	PM	PMCG		Trt	Year	Trt * Year
BW, kg								
Initial	430	430	430	428	4.1	0.98	<0.01	1.00
Middle	463	464	459	464	4.5	0.80	<0.01	0.96
Final	481	490	481	484	4.7	0.49	<0.01	0.97
Total gain	51.5	59.6	50.8	55.6	3.3	0.21	<0.01	0.50
ADG, kg/d								
Period 1	0.99	0.99	0.84	1.04	0.06	0.16	<0.01	0.28
Period 2	0.72	1.00	0.85	0.92	0.10	0.20	<0.01	0.15
Total ADG	0.86	0.99	0.85	0.97	0.06	0.17	<0.01	0.20

Total gain and ADG differed by year (*P* < 0.01; Table 3). Steers in both 2014 and 2016 exhibited greater (*P* < 0.01) total body weight gain compared to steers in 2015 (59.1, 63.1, and 41.0 kg, respectively). However, steers in 2016 had a greater (*P* < 0.01) ADG in comparison to steers in 2014 (1.25 vs. 0.84 kg/d, respectively), and both of these were greater (*P* < 0.01) than in 2015 (0.65 kg/d). Greater ADG in 2014 compared to 2015 was likely the result of more favorable weather conditions (Harmon et al., 2019), which provided soil moisture for greater forage production and less heat stress for the calves in the first year. Though the weather conditions in 2016 were less favorable than 2014 or 2015, the superior ADG in 2016 may have been the result of compensatory gain for steers that entered the trial at a lighter weight than the steers in the other 2 yr (437, 440, and 411 kg in 2014, 2015, and 2016, respectively). The compensatory gain of calves in 2016 was evident during the first weigh period, with steers in 2016 having a greater (*P* < 0.01) ADG in the first period of the trial than for steers in 2014 and 2015 (1.15, 0.85, and 0.89 kg/d, respectively).

Although the main effect of year was significant for all measures of growth at each time point and period, there were no effects of treatment within or between years (*P* > 0.15). Across years, at the end of the grazing trial, final BW, total BW gain, and total ADG did not differ (*P* > 0.17) among treatments. In a comparison of 2 sorghum × sudangrass varieties, McCuistion et al. (2011) found similar 56 d ADG to those reported in this study. In their study, steers grazing brown midrib sorghum × sudangrass had an ADG of 0.96 kg/d while steers grazing a nonbrown midrib sorghum × sudangrass variety exhibited an ADG of 0.82 kg/d. The authors suggested that the trends seen in ADG were the result of differences in forage nutritional composition associated with digestibility and the brown midrib gene; however, those differences were not observed in the current study.

Schmidt et al. (2013) reported steers grazing pearl millet had an ADG that exceeded 0.56 kg/d but was less than that for steers grazing bermudagrass (0.76 kg/d). McCartor and Rouquette (1977) reported a wide range in ADG of steers grazing pearl millet, from 0.27 to 1.01 kg, depending on stocking rate and forage availability. Ball et al. (2002) suggested that pearl millet was only high in nutritive value while in the immature state, explaining the range in gains in the literature. Though the addition of crabgrass to the pearl millet in the current study did not result in an improvement in total or average daily gains, the nutritive value of crabgrass (Dalrymple, 2001; Ogden et al., 2005) did not negatively impact it, either. Additionally, having crabgrass as part of the species mix may have benefits other than animal performance, such as aiding in the reduction of weed pressures between pearl millet plants and increasing the percentage of desirable species in the sward (Harmon et al., 2019).

### Gain Per Hectare

Differences in total live weight gain per hectare of warm-season annual pastures are presented in Table 4. Gain per hectare was affected by treatment (*P* = 0.02) and year (*P* < 0.01), and a tendency was detected by the interaction of treatment and year (*P* = 0.06). Sorghum × sudangrass (SS) had greater (*P* = 0.02) gains per hectare than PM and PMCG, with BMR as an intermediate (246, 181, 188, and 226, respectively). As expected, the 2014 grazing year, where moisture was plentiful, resulted in greater (*P* < 0.01) gains per unit of land than 2016 (272 vs. 207 kg/ha, respectively), which was greater (*P* < 0.01) than 2015 (152 kg/ha). A forage treatment effect was detected in 2015 (*P* < 0.01) but not in 2014 (*P* = 0.12) or 2016 (*P* = 0.25). In 2015, BMR had greater (*P* < 0.02) total gains than SS, PMCG, and PM. In that year, SS was greater (*P* < 0.03) than PM, but PMCG was an intermediate between the two. In this study, total gain per hectare was less than what was reported by Hill et al. (1993) in steers grazing Tifton 78 (4.67 kg/ha/d) and Tifton 85 (6.84 kg/ha/d) bermudagrass pastures for 169 d. However, the 3-yr average total precipitation during their grazing experiment was greater than what was observed in the current study, which potentially limited the performance of pastures. Comparably, Hill et al. (1999) reported that during a 2-yr pearl millet grazing trial, total gain was 6.46 and 5.94 kg/ha/d, with average total monthly precipitation during the 84 d trial of 77.7 and 91.9 mm, respectively. In this study, average total monthly precipitation during the trial was 119, 87, and 45 mm for 2014, 2015, and 2016, respectively. Thus, the greater total gain per unit of land found in their study may be reflective of the improved distribution of timely rainfall events in combination with higher inputs of nitrogen fertilizer (252 kg/ha of N).

**Table 4.** Least squares means for total live weight gain per hectare of sorghum × sudangrass (SS), brown midrib sorghum × sudangrass (BMR), pearl millet (PM), and pearl millet and crabgrass mixture (PMCG) pastures in 2014, 2015, and 2016 at the University of Georgia, Department of Animal and Dairy Science Eatonton Beef Research Unit in Eatonton, GA

Year^1^	Forage treatment, kg/ha				SEM	*P*-value
	SS	BMR	PM	PMCG		
2014	375	257	207	249	45.0	0.12
2015	156^b^	189^a^	126^c^	136^bc^	8.3	<0.01
2016	207	233	210	180	17.0	0.25

^a–c^Means within a row without a common superscript differ (*P* < 0.05).

^1^Grazing days: 2014 = 70 d; 2015 = 63 d; 2016 = 56 d.

### Ultrasound Measurements

Ultrasonically measured carcass composition traits are presented in Table 5. Main effect of treatment was not significant (*P* > 0.16) for any measured variable on any scan date. As expected, both uLM and uRFT increased by date (*P* < 0.01) and were greatest (*P* < 0.01) for steers in 2014 and 2015 compared to 2016 (63.0, 61.8, and 58.5 cm^2^, respectively, for uLM and 0.58, 0.56, and 0.40 cm, respectively, for uRFT). Similarly, uFT also increased by scan date (*P* < 0.01) and was greatest (*P* < 0.01) for steers in 2014 compared to 2015 and 2016 (0.54, 0.49, and 0.45 cm, respectively). Increases in uLM, uRFT, and uFT as days on pasture increased is reflective of the nutritive value of warm-season annual grass pastures and their ability to exceed nutrient requirements of growing and finishing cattle (NRC, 2000). Intramuscular fat (uIMF) decreased (*P* < 0.01) between the initial scan date and the middle scan date and increased (*P* < 0.01) from the middle to the final scan date for steers grazing SS, BMR, PM, and PMCG pastures. The observed relationship may be a result of the unproportioned and rapid increase in LM area compared to IMF and thus altering the overall ratio of the 2 components (Owens et al., 1993).

**Table 5.** Least squares means for ultrasound measurements of forage-finished steers pastured on sorghum × sudangrass (SS), brown midrib sorghum × sudangrass (BMR), pearl millet (PM), or a mixture of pearl millet and crabgrass (PMCG) during a forage-finishing trial conducted during the summers of 2014 to 2016 at the UGA Animal and Dairy Science Department’s Beef Research Unit near Eatonton, GA

Item/time point^1^	Forage treatment				SEM	Effect		
	SS	BMR	PM	PMCG		Trt	Year	Trt * Year
uLM, cm^2^								
Initial^2^	57.7	57.0	57.6	55.4	1.04	0.28	0.01	0.79
Middle^3^	62.3	61.5	64.7	60.7	1.28	0.16	<0.01	0.65
Final^4^	65.6	63.4	63.3	64.1	1.10	0.40	<0.01	0.69
uFT, cm								
Initial^2^	0.39	0.41	0.38	0.36	0.03	0.45	0.06	0.80
Middle^3^	0.50	0.54	0.52	0.50	0.03	0.77	0.06	0.61
Final^4^	0.55	0.59	0.62	0.57	0.04	0.54	<0.01	0.72
uIMF, %								
Initial^2^	3.55	3.43	3.59	3.59	0.10	0.66	0.03	0.07
Middle^3^	3.17	3.28	3.24	3.37	0.10	0.53	<0.01	0.14
Final^4^	3.75	3.62	3.85	3.65	0.12	0.50	0.58	0.16
uRFT, cm								
Initial^2^	0.43	0.44	0.46	0.41	0.02	0.47	<0.01	0.28
Middle^3^	0.54	0.50	0.51	0.51	0.03	0.90	<0.01	0.41
Final^4^	0.57	0.62	0.61	0.55	0.03	0.41	<0.01	0.97

^1^uLM = ultrasound measurement of the longissimus muscle area at the 12th to 13th rib juncture; uFT = ultrasound measurement of the 12th rib fat thickness; uIMF = ultrasound measurement of the percent LM intramuscular fat; uRFT = ultrasound measurement of the rump fat thickness.

^2^Initial = June 25, 2014; June 25, 2015; and June 29, 2016.

^3^Middle = July 29, 2014; July 29, 2015; and August 2, 2016.

^4^Final = September 3, 2014; August 27, 2015; and August 31, 2016.

### Carcass Characteristics

Means of treatment for carcass characteristics associated with yield grade are reported in Table 6. Forage treatment did not have an impact on carcass characteristics or calculated yield grade. Though SS tended to have a greater dressing percentage (*P* = 0.06) and carcasses from the PM steers tended to have larger ribeye area (*P* < 0.10), these differences were relatively minor and of questionable practical significance. However, the effect of year on carcass characteristics and yield grade was significant (*P* < 0.01) for all the measured variables, with the exception of shrunk BW and fat thickness (*P* > 0.31). Steers finished in 2014 and 2015 had a greater HCW (*P* < 0.01) and dressing percent (*P* < 0.01) than steers in 2016. This is likely the result of extreme drought and heat stress the cattle experienced in 2016. Carcasses from steers finished in 2014 had a greater LM area (*P* < 0.01) than carcasses in 2015 and 2016 (73.2, 67.7, and 65.8 cm^2^, respectively). The greater LM area in 2014 compared to 2015 is likely the result of the aforementioned greater ADG in 2014. However, the difference between 2014 and 2016 in measures of LM area is likely the result of a difference between the years in live weight and hot carcass weight.

**Table 6.** Least squares means for yield-associated carcass characteristics for forage-finished steers pastured on sorghum × sudangrass (SS), brown midrib sorghum × sudangrass (BMR), pearl millet (PM), or a mixture of pearl millet and crabgrass (PMCG) during a forage-finishing trial conducted during the summers of 2014 to 2016 at the UGA Animal and Dairy Science Department’s Beef Research Unit near Eatonton, GA

Item	Forage treatment				SEM	Effect		
	SS	BMR	PM	PMCG		Trt	Year	Trt * Year
Shrunk BW, kg	459	470	462	463	4.43	0.37	0.31	0.81
HCW, kg	267	268	267	265	3.29	0.91	<0.01	0.62
Dressing %	58.0	57.0	57.8	57.2	0.30	0.06	<0.01	0.51
LM area, cm^2^	66.9	69.7	71.5	67.5	1.40	0.08	<0.01	0.58
LM area/kg LWT	14.6	14.9	15.5	14.6	0.30	0.10	<0.01	0.56
LM area/kg HCW	25.1	26.1	26.9	25.5	0.54	0.08	<0.01	0.61
KPH, %	1.5	1.6	1.5	1.4	0.11	0.34	<0.01	0.08
Fat thickness, cm	0.49	0.51	0.50	0.50	0.04	0.99	0.77	0.82
Yield grade^1^	2.2	2.1	2.0	2.2	0.10	0.39	<0.01	0.63

^1^Yiled grade calculated by 2.5 + (2.50 * adj. fat thickness, in) + (0.20 * %KPH) + (0.0038 * hot carcass weight, lbs) − (0.32 * longissimus area, in^2^).

Much of the current literature has focused on utilizing cool-season grasses and legumes because of their high nutritive value and resulting impact on animal performance and carcass quality. However, Neel et al. (2007) reported that steers finished on a mix of cool-season grass and legume pastures to have a HCW of 247 kg and was lighter than what was found in this study, even though final BW between the 2 studies was comparable. Furthermore, the LM area reported for the pasture-finished steers in that study was 66 cm^2^, which is similar to slightly less than the range of LM area found in the SS, BMR, PM, and PMCG treatments. There was no difference (*P* = 0.34) attributed to forage source for KPH; however, steers finished in 2014 and 2015 had a lower (*P* < 0.01) percentage of KPH than steers from 2016 (1.4, 1.3, and 1.9%, respectively). Though not impacted by forage treatment, yield grade was lower (*P* < 0.02) for carcasses in 2014 compared to 2015 or 2016 (1.9, 2.2, and 2.3, respectively) and may reflect differences seen in steer HCW and thus LM area between years. The lack of forage-finishing treatment effect on yield grade is consistent with other reports (Schmidt et al., 2013).

Carcass characteristics associated with carcass quality grade including maturity, color, firmness, and texture did not differ (*P* > 0.11) among forage species (Table 7). Year had a significant effect on many of the variables tested, suggesting that environment is capable of influencing finishing performance in forage-fed cattle. Steers finished in 2014 displayed an increase (*P* < 0.01) in lean maturity over those finished in 2015 and 2016 (211, 169, and 166, respectively) while overall maturity was lower (*P* < 0.01) in 2015 compared to the other 2 trial years (143 vs. 154 in 2014 and 153 in 2016). These results again reiterate the advancement in growth at the initiation of the trial for steers in year 2014. Skeletal maturity was greatest (*P* < 0.01) for steers in year 2016, followed by 2015 and least for 2014 (173, 147, and 132 maturity, respectively). Steers in 2015 exhibited (*P* < 0.01) a slightly darker red lean color compared to the moderately dark red color seen in steers from 2014 and 2016 (5.19, 4.16, and 4.00, respectively). Additionally, a greater (*P* < 0.01) subjective yellow external fat color, which has been characteristically associated with grass-fed beef, was more visible in 2015 and 2016 steers compared to 2014 (5.16, 5.25, and 3.25, respectively). Redness values (a^*^) were greater in fat (*P* < 0.01) and lean (*P* < 0.01) in 2015 than 2014 or 2016 (9.57, 8.03, and 8.40, respectively; and 30.66, 29.62, and 29.11, respectively). Yellowness values (b^*^) for fat were least (*P* < 0.01) in carcasses from 2014 steers compared to 2015 and 2016 (22.03, 25.03, and 24.70, respectively) while lean yellowness values were greatest (*P* < 0.01) for those harvested in 2015 vs. 2014 and 2016 (22.75, 21.49, and 20.98, respectively). There was a tendency (*P* > 0.06) for year to effect carcass firmness (1.91, 1.63, and 2.13, for years 2014, 2015, and 2016, respectively) but not texture. Steers in 2014 had a greater marbling score than steers in 2015 and 2016 (386, 349, and 348, respectively); however, all steers finished with a marbling score of slight.

**Table 7.** Least squares means for quality-associated carcass characteristics for forage-finished steers pastured on sorghum × sudangrass (SS), brown midrib sorghum × sudangrass (BMR), pearl millet (PM), or a mixture of pearl millet and crabgrass (PMCG) during a forage-finishing trial conducted during the summers of 2014 to 2016 at the UGA Animal and Dairy Science Department’s Beef Research Unit near Eatonton, GA

Item	Forage treatment				SEM	Effect		
	SS	BMR	PM	PMCG		Trt	Year	Trt * Year
Lean maturity^1^	175	185	187	181	4.98	0.38	<0.01	0.80
Skeletal maturity^1^	152	150	152	149	2.89	0.86	<0.01	0.45
Overall maturity^1^	147	151	152	149	2.74	0.57	<0.01	0.88
Subjective lean color^2^	4.3	4.6	4.6	4.3	0.21	0.39	<0.01	0.87
Subjective fat color^3^	4.6	4.7	4.5	4.5	0.21	0.90	<0.01	0.59
Objective lean color								
L^*4^	36.77	37.24	36.37	37.29	0.49	0.51	0.01	0.62
a^*5^	29.98	30.38	29.10	29.72	0.38	0.12	<0.01	0.24
b^*6^	21.92	22.38	21.03	21.65	0.41	0.13	<0.01	0.14
Objective fat color								
L^*4^	80.11	80.34	79.73	80.18	0.44	0.80	0.31	0.67
a^*5^	8.75	8.71	8.89	8.32	0.34	0.69	<0.01	0.13
b^*6^	23.85	24.18	24.49	23.17	0.69	0.56	<0.01	0.51
Firmness^7^	1.7	2.1	1.9	1.8	0.17	0.47	0.06	0.71
Texture^8^	1.3	1.3	1.6	1.4	0.12	0.15	0.27	0.54
Marbling^9^	364	352	370	359	8.39	0.47	<0.01	0.23

^1^100 = A00; 200 = B00.

^2^1 = extremely dark red; 2 = very dark red; 3 = dark red; 4 = moderately dark red; 5 = slightly dark red; 6 = cherry red; 7 = moderately bright cherry red; 8 = light cherry red.

^3^1 = white; 2 = creamy white; 3 = slightly yellow; 4 = moderately yellow; 5 = yellow.

^4^Measurement of lightness; 0 = darker; 100 = lighter.

^5^Measurement of green to red; greater value indicates increased redness.

^6^Measurement of blue to yellow; greater value indicates increased yellowness.

^7^1 = very firm; 2 = firm; 3 = slightly firm; 4 = slightly soft; 5 = soft.

^8^1 = very fine; 2 = fine; 3 = slightly fine; 4 = slightly coarse; 5 = coarse.

^9^300 = Slight00; 400 = Small00; 500 = Modest00.

Marbling scores in this study were less than what was reported by Schmidt et al. (2013), who found that steers finished on pearl millet and cowpea had marbling scores of 473 and 513, representing slight and small degrees of marbling, respectively. The differences in quality grade between years in this study can be attributed to the impact temperature and heat stress has on carcass composition and is similar to what others have reported. Mitlöhner et al. (2002) found that heifers provided with shade had a greater quality grade than unshaded heifers. Kreikemeier et al. (1998) reported a similar impact on quality grade for cattle harvested during the summer months compared to those harvested in milder conditions. Additionally, time on feed may explain differences reported in year-to-year variation of marbling score and quality grade.

## SUMMARY AND CONCLUSIONS

There is very little information on the utilization of warm-season annual grasses in forage-finishing beef systems. Much of the literature has focused on utilizing cool-season grasses and legumes because of their impeccable forage quality and resulting impact on animal performance and carcass quality. With the increase in demand for forage-finished beef products and the opportunity to produce a high-quality product, alternative forage systems must be considered for summer forage finishing of beef in the southeastern United States. In this study, steers grazing SS, BMR, PM, and PMCG pastures all performed similarly during summer forage finishing. Environmental impacts on forage had a greater impact on steer performance and carcass composition than forage treatments. Forage and steer performance were greater in years with ample moisture and moderate temperatures. However, even during years where moisture was limited and temperatures were above average, all forage treatments induced a gain response in cattle, indicating SS, BMR, PM, and PMCG may be used for forage finishing in a range of climatic regions. Forage treatment did not affect live animal performance, or carcass characteristics used to determine both yield and quality grades in cattle. Producers can utilize these forage systems interchangeably in forage-finishing operations, without negatively impacting the final beef product.
